# NAMI-A and KP1019/1339, Two Iconic Ruthenium Anticancer Drug Candidates Face-to-Face: A Case Story in Medicinal Inorganic Chemistry

**DOI:** 10.3390/molecules24101995

**Published:** 2019-05-24

**Authors:** Enzo Alessio, Luigi Messori

**Affiliations:** 1 Department of Chemical and Pharmaceutical Sciences, University of Trieste, Via L. Giorgieri 1, I-34127 Trieste, Italy; 2 Department of Chemistry ‘Ugo Schiff’, University of Florence, Via della Lastruccia 3-13, I-50019 Sesto Fiorentino, Italy

**Keywords:** anticancer, antimetastasis, uptake, protein binding, ruthenium, clinical study, biodistribution, activation, aquation

## Abstract

NAMI-A ((ImH)[*trans*-RuCl_4_(dmso-S)(Im)], Im = imidazole) and KP1019/1339 (KP1019 = (IndH)[*trans*-RuCl_4_(Ind)_2_], Ind = indazole; KP1339 = Na[*trans*-RuCl_4_(Ind)_2_]) are two structurally related ruthenium(III) coordination compounds that have attracted a lot of attention in the medicinal inorganic chemistry scientific community as promising anticancer drug candidates. This has led to a considerable amount of studies on their respective chemico-biological features and to the eventual admission of both to clinical trials. The encouraging pharmacological performances qualified KP1019 mainly as a cytotoxic agent for the treatment of platinum-resistant colorectal cancers, whereas the non-cytotoxic NAMI-A has gained the reputation of being a very effective antimetastatic drug. A critical and strictly comparative analysis of the studies conducted so far on NAMI-A and KP1019 allows us to define the state of the art of these experimental ruthenium drugs in terms of the respective pharmacological profiles and potential clinical applications, and to gain some insight into the inherent molecular mechanisms. Despite their evident structural relatedness, deeply distinct biological and pharmacological profiles do emerge. Overall, these two iconic ruthenium complexes form an exemplary and unique case in the field of medicinal inorganic chemistry.

## 1. KP1019 and NAMI-A, Two Structurally Similar Ruthenium Complexes for Cancer Treatment: Introductive Remarks

**Figure 1:**
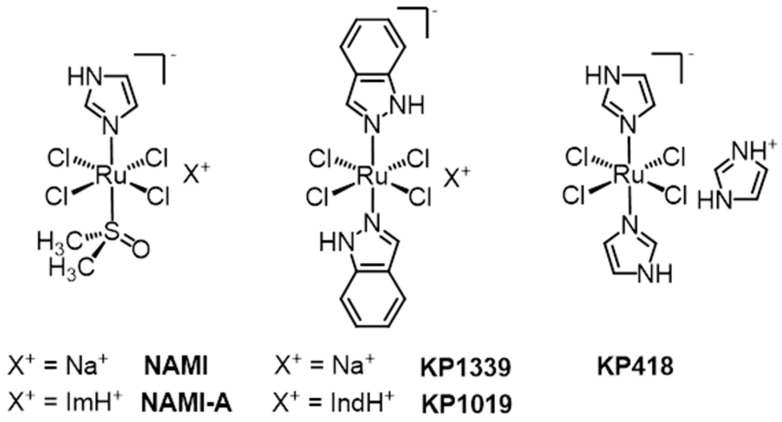
Schematic structures of NAMI-A ((ImH)[*trans*-RuCl_4_(dmso-S)(Im)], Im = imidazole), KP1019/KP1339 (KP1019 = (IndH)[*trans*-RuCl_4_(Ind)_2_], Ind = indazole; KP1339 = Na[*trans*-RuCl_4_(Ind)_2_]), and KP418 (imidazolium *trans*-bis-imidazoletetrachlororuthenate(III), (ImH)[*trans*-RuCl_4_(Im)_2_]). KP1019 is sometimes also called FFC14, or FFC14a, or FFC14A. The sodium salt of KP1019, besides KP1339, is also called KP-1339, or NKP1339, or—more recently—IT-139. Originally, the imidazole complex KP418 was called ICR.

Two structurally related Ru(III) coordination compounds, known as NAMI-A ((ImH)[*trans*-RuCl_4_(dmso-S)(Im)], Im = imidazole) and KP1019/KP1339 (KP1019 = (IndH)[*trans*-RuCl_4_(Ind)_2_], Ind = indazole; KP1339 = Na[*trans*-RuCl_4_(Ind)_2_], i.e., the sodium salt of KP1019, Figure 1), have eventually reached the stage of clinical evaluation in humans, opening the way to large expectations for a new class of metal-based anticancer drugs. This review is intended to analyze comparatively the main features of these two putative drugs almost 30 years after their discovery; within the review, the current understanding of their mechanisms of action and the perspectives for clinical application are illustrated. In the course of their development and characterization, several detailed review articles have focused on KP1019 [1,2,3] or NAMI-A [4,5,6,7,8,9,10] or both [11,12,13,14,15,16,17,18,19,20,21,22] (and other metal compounds), to which the interested reader is referred.

Briefly, we can say here that KP1019 and NAMI-A were initially discovered as a consequence of the intense synthetic work carried out in the field of anticancer metal complexes after the clinical approval of cisplatin in 1978. Pioneering work on Ru complexes was initially conducted by M.J. Clarke et al. in the 1980s, who investigated simple Ru(III) chloroammine compounds, such as *fac*-[RuCl_3_(NH_3_)_3_] and *cis*-[RuCl_2_(NH_3_)_4_]Cl [23], which were directly modeled on the basis of cisplatin. Remarkably, in 1986, B.K. Keppler et al. reported for the first time on the antitumor activity of an innovative water-soluble anionic Ru(III) complex—i.e., imidazolium *trans*-bis-imidazoletetrachlororuthenate(III), (ImH)[*trans*-RuCl_4_(Im)_2_] (Im = imidazole), which was later labeled as KP418 (Figure 1), against P388 leukemia and B16 melanoma in BDF_1_ mice [24]. In a way, KP418 is the immediate precursor of KP1019 and, in turn, of NAMI-A. Notably, KP418 manifested a high efficacy against an autochthonous model of colorectal cancer. The tumor inhibiting effect was even better than that of cyclophosphamide, cisplatin, or 5-fluorouracil, which were used as reference compounds. Comparable results, which had a tumor growth inhibition exceeding 90%, were later obtained with the less toxic indazole (Ind) analogue, (Hind)[*trans*-RuCl_4_(Ind)_2_] (KP1019, Figure 1) [25], which was later replaced by the more soluble sodium salt Na[*trans*-RuCl_4_(Ind)_2_] (KP1319/NKP1339/FCC14A/IT-139, Figure 1), which was obtained from KP1019 in a two-step cation exchange via the tetramethylammonium salt [26]. It is worth stressing that the investigated tumor model is not sensitive to clinically established antineoplastic agents, including cisplatin, with the exception of the 5-fluorouracil/leucovorin combination therapy, which shows moderate activity.

The exciting results reported by Keppler et al. on the Ru(III)-azole complexes triggered the development in the early 1990s of another class of structurally related Ru(III)-dmso compounds. G. Mestroni and E. Alessio first prepared the Ru(III)-dmso intermediate X[*trans*-RuCl_4_(dmso-S)_2_] (X^+^ = (dmso)_2_H^+^, Na^+^, NH_4_^+^), which has an obvious structural similarity with the anticancer active *trans*-azole Ru(III) complexes (KP-type compounds) described above [27]. Although per se unsuited for biological tests, Na[*trans*-RuCl_4_(dmso-S)_2_] turned out to be an excellent precursor for compounds of the general formula Na[*trans*-RuCl_4_(dmso-S)(L)] (where L = NH_3_, azole or pyridine), which showed a greater stability in aqueous solution [28]. Tests performed by Sava et al. on solid metastasizing tumors in mice soon evidenced that these compounds induced a remarkable reduction of lung metastasis formation, which was significantly greater than the unimpressive reduction of primary tumor growth [29,30]. Surprisingly, the antimetastatic effect was more pronounced with low doses given daily than with large doses given with drug-free intervals. Then, the water-soluble imidazole complex Na[*trans*-RuCl_4_(dmso-S)(Im)] (NAMI, Figure 1) was selected for further investigations. NAMI was later replaced by the corresponding imidazolium salt, (ImH)[*trans*-RuCl_4_(dmso-S)(Im)], called NAMI-A, that showed improved preparation, a greater stability in the solid state, and a better analytical profile, while maintaining a good solubility in water.

Interestingly, both the KP-type and the NAMI-A-type compounds did not go through the usual pre-screening of in vitro cytotoxicity against cancer cell lines, but were instead investigated immediately on animal models. This unusual procedure was instrumental to their further developments: in fact, neither one of these structurally similar Ru(III) complexes is particularly cytotoxic (see also below) [31].

## 2. KP1019 and NAMI-A: Structural and Solution Chemistry; Biomolecular Interactions

A detailed comparative description and interpretation of the chemical features and the chemical behavior in solution of NAMI-A and KP1019 is strictly required to better understand their respective biological profiles. Extensive literature is now available on these topics [1,2,3,4,5,6,7,8,9,10,11,12,13,14,15,16,17,18,19,20,21,22]. The main features of these two structurally similar complexes will be briefly described below.

### 2.1. Structural and Solution Chemistry

Both NAMI and NAMI-A share the same pseudo-octahedral ruthenium(III) center surrounded by four chloride ligands in the equatorial plane. One S-bonded dmso (dmso-S) and one imidazole occupy the two axial positions. The negative charge is neutralized by Na^+^ in NAMI and by the imidazolium cation in NAMI-A. NAMI-A is perfectly stable in the solid state, whereas it undergoes relatively fast hydrolytic processes in aqueous solution that are strongly pH-dependent [5,6,32,33]. At 37 °C and physiological pH, the parent complex disappears from the solution within ca. 15 min due to rapid chloride and dmso hydrolysis, leading to the formation of dark-green poly-oxo species and eventually to a black precipitate. On the contrary, the complex is remarkably more stable at mildly acidic pH (3.0–6.0) and in pure water (pH ca. 5.5), where slow dmso hydrolysis only occurs. Interestingly—and in agreement with a dissociative aquation mechanism—NAMI-A-type complexes bearing azole ligands that are less basic than imidazole, such as pyrazole and thiazole, are more stable than NAMI-A in slightly acidic aqueous solution [34].

The presence of dmso-S in the coordination sphere confers a relatively high reduction potential to NAMI-A: *E*° + 235 mV versus NHE (Normal Hydrogen Electrode) [28,35,36]. As a consequence, NAMI-A at pH 7.4 and 25 °C is quantitatively and nearly instantaneously reduced to the corresponding dianionic Ru(II) species [*trans*-RuCl_4_(dmso-S)(Im)]^2–^ by the addition of stoichiometric amounts of biologically relevant reducing agents, such as ascorbic acid (AsH) or glutathione (GSH) [35,37].

The geometry of the Ru(III) anion in KP1019 is similar to that of NAMI-A, with four equatorial chloride ligands and two axial indazole ligands. KP1019 is stable in the solid state, and shows a moderate solubility in water, in which it is significantly more stable than NAMI-A. In fact, in aqueous solution at 25 °C, the slow exchange of one chloride ligand for water (ca. 2% per hour) occurs [38], generating the corresponding neutral complex *mer*,*trans*-RuCl_3_(Ind)_2_(OH_2_), which has been isolated and structurally characterized [39]. Similarly to NAMI-A, KP1019 hydrolyzes faster upon increasing the pH: at 37 °C, the half-life of the complex is 5.4 h in phosphate buffer at pH 6.0, whereas it is less than 0.5 h at pH 7.4, where the release of the indazole ligands also occurs [38]. The formation of a precipitate within minutes from a fresh solution of KP1019, dependent on concentration, pH, and temperature, was often reported [38,40]. In phosphate buffer, at pH 7, KP1019 has a lower redox potential compared to NAMI-A (*E*° = +30 mV versus NHE, measured on the more soluble sodium salt KP1339) [36]. Keppler et al. demonstrated that both ascorbic acid and glutathione are capable of reducing the *trans*-[RuCl_4_(Ind)_2_]^–^ anion, even though at a slower rate than NAMI-A: in phosphate-buffered solution, the complete reduction of KP1019 required anywhere from minutes (AsH) to hours (GSH), even when GSH or AsH are in slight excess [41]. In parallel, the precipitation of uncharacterized species was observed.

### 2.2. Biomolecular Interactions

The interactions of NAMI-A and KP1019 with a few biologically relevant molecules have been studied in detail through a variety of methods paying particular attention, on one hand, to DNA and to short single or double-stranded oligonucleotides—more recently to RNA—and on the other hand, to model and transport proteins.

#### 2.2.1. Nucleic Acids

An important study that analyzes the interactions of NAMI-A and KP1019 with DNA molecules in a cell-free medium was contributed by Brabec et al. [42], and recently included in a dedicated review paper [43]. The modifications of natural DNA were characterized through a battery of biophysical methods. Overall, the results indicated that the two Ru compounds are able to coordinate irreversibly to DNA; however, their DNA binding mode appears to be profoundly different from that of cisplatin. NAMI-A binds to DNA in vitro considerably faster than KP1019 and also cisplatin, which is in accord with its greater kinetic reactivity; it also forms bifunctional intrastrand adducts on double-helical DNA that are capable of terminating RNA synthesis in vitro. Conversely, the ability of KP1019 to form such adducts is markedly lower. Even though the binding of both NAMI-A and KP1019 affects the DNA structure, the conformational changes induced by ruthenium compounds are usually smaller than those of platinum drugs, resulting in a less severe DNA distortion and damage. Later, Keppler et al. used advanced mass spectrometry methods to describe the interactions of the two complexes with oligonucleotides [44]. Specifically, the binding of KP1019 and NAMI-A toward different double-stranded oligonucleotides was probed by electrospray ionization mass spectrometry and compared with that of the platinum drugs cisplatin, carboplatin, and oxaliplatin. Notably, the extent of adduct formation decreased in the following order: cisplatin > oxaliplatin > NAMI-A > KP1019. The binding sites of these metallodrugs on the oligonucleotides were identified using top–down tandem mass spectrometry; in all cases, a strong preference for direct binding to guanine residues was highlighted. A similar result was obtained in a more recent study that compared the interactions of NAMI-A and of the pyridine analog (pyH)[*trans*-RuCl_4_(dmso-S)(py)] (nicknamed AziRu) toward both single-stranded and duplex oligonucleotides [45]. In both cases, the formation of stable adducts between the two complexes and guanine-containing oligomers was observed. Interestingly, the oligonucleotide structure incorporated the {Ru(Im)} fragment when treated with NAMI-A, but a naked Ru ion when treated with AziRu.

The first study on the interactions of NAMI-A with RNA was reported in 2011 [46]. When yeast (*Saccharomyces cerevisiae*) was treated with 150-μM and 450-μM NAMI-A solutions, a significant dose-dependent accumulation of Ru in the extracted cellular RNA was observed. In vitro, model RNA (and DNA) oligonucleotides were found to bind aquated derivatives of NAMI-A. The enhanced binding of Ru species occurred under reducing conditions (ascorbic acid). Very recently, a NAMI-A/KP1019 comparative study was performed on a more structurally complex model, tRNA, which is the most abundant, stable, and soluble form of RNA in the cytosol [47]. In fact, more sophisticated nucleic acid structures such as tRNA tertiary structures provide a variety of binding pockets that are not accessible to short oligonucleotides, where interactions of metal fragments with specific bases prevail. KP1019 was found to bind more tightly than NAMI-A to tRNA. In addition, contrary to what has been previously found with oligonucleotides [46], the reduction of both compounds to Ru(II) resulted in a significant decrease in binding. Whereas the binding of NAMI-A is best explained by electrostatic interactions, KP1019 is believed to intercalate—through the relatively large indazole ligands—in the π-stacks of tRNA within structurally complex binding pockets.

#### 2.2.2. Proteins

The interactions of NAMI-A and KP1019 with proteins have been intensely investigated. Notably, early studies were focused on their interactions with the major serum proteins: i.e., serum albumin (HSA), which is by far the most abundant protein in the plasma (ca. 600 μM), and serum transferrin (HSTf, whose concentration is ca. 15–20 times lower than HSA). In particular, interest in serum transferrin was specifically driven by the consideration that the chemical behavior of Ru(III) might be somehow similar to that of Fe(III). Accordingly, it was proposed, mainly by Keppler et al., that the transport mechanisms of iron might be exploited by the non-physiological ruthenium species as a smart way to enter cells, according to a “Trojan horse” strategy [3]. In addition, as cancer cells usually require a far larger amount of iron than healthy cells and express a greater number of transferrin receptors [48,49,50], it was suggested that such transferrin-mediated uptake could impart some selectivity for cancer cells to ruthenium compounds. The studies, although initially centered on the interactions with serum transferrin, were subsequently extended to the major serum protein, i.e., serum albumin, for which other internalization mechanisms are available.

We like to stress that some relevant contributions to the understanding of the interactions of KP1019 and NAMI-A with serum proteins came from the groups of Lay, Walsby, and Harris, using a variety of spectroscopic methods, in particular XAS, ESR, and ENDOR [51,52,53,54,55]. Notably, both Ru compounds were shown to bind very rapidly to HSA in a noncovalent manner, followed by coordination to protein side chains after ligand exchange. Some evidence also suggested that the adducts formed between NAMI-A and HSA are still able to produce important pharmacological effects at the cellular level, and are potentially linked to its antimetastatic activity, such as increased cell adhesion to the substrate, reduced cell motility, and the decreased ability of cells to penetrate into collagen gels [56,57].

Yet, the general role and importance of the interactions of these ruthenium drugs with serum proteins have not been validated and remain elusive and highly controversial, as it will be discussed later.

On the other hand, a few recent studies were specifically aimed to model, at the atomic level, the interactions of a few putative Ru drugs with standard proteins. These studies, relying on a combined X-ray diffraction and ESI-MS approach, elucidated the nature of the occurring interactions and the general mode of Ru binding. These aspects are treated exhaustively and competently in a recent structural review by Merlino [58], to which the interested reader is referred; we will consider here just a few key aspects emerging from those investigations.

##### 2.2.2.1. NAMI-A

**Figure 2:**
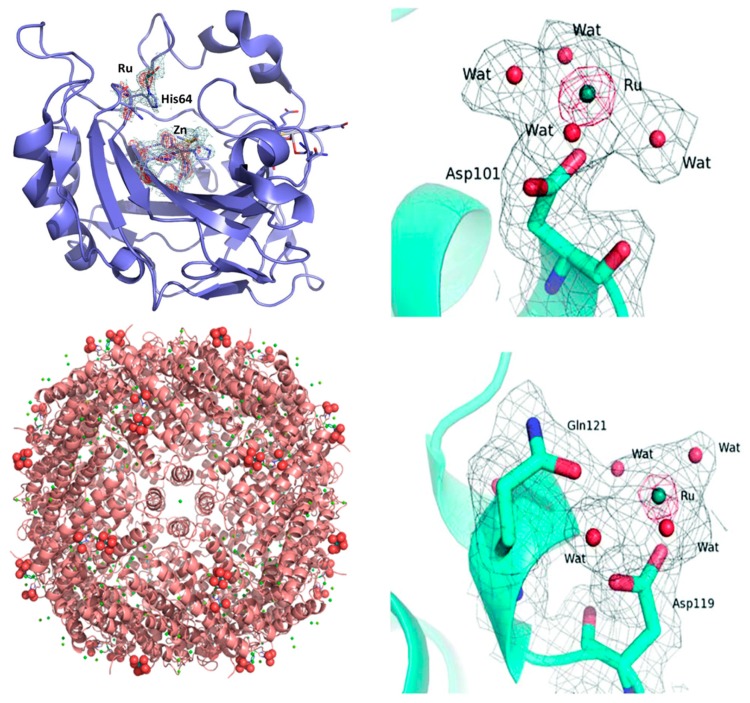
Left: The adduct of NAMI-A with hen egg white lysozyme (HEWL). (top) Ru binding site close to Asp101. (bottom) Ru binding site close to Asp119. Reproduced from Ref. 59 by permission of The Royal Society of Chemistry. Top right: The adduct of NAMI-A with carbonic anhydrase (hCAII). Detail of the Ru center interactions with residues His 64. The oxygen atoms from water molecules are represented as red spheres. Reproduced from Ref. [60] with permission from Elsevier. Bottom right: Ribbon representation of the overall structure of the NAMI-A/HuHf (human ferritin) adduct. The side chain of His105 is shown as a ball and stick, while Ru and the water molecules completing the metal coordination sphere are shown as spheres. Reproduced from Ref. [61] by permission of The Royal Society of Chemistry.

The interactions of NAMI-A with the model proteins lysozyme [59], carbonic anhydrase [60], and—very recently—human H-chain ferritin [61] have been investigated in detail; now, there is sufficient evidence to illustrate the underlying modes of interaction. In the case of hen egg white lysozyme (HEWL), the X-ray structure showed that NAMI-A behaves as an “ultimate prodrug” [10], losing all its original ligands during the soaking process: the resulting naked ruthenium ions interact with the protein through the formation of coordinative bonds to the carboxylate groups of two distinct aspartate residues, i.e., Asp 101 and Asp 119 (Figure 2). Similar results were obtained upon solving the crystal structure of the adducts formed by NAMI-A with carbonic anhydrase (hCAII) and human ferritin (HuHf). Again, a naked ruthenium ion was detected coordinatively bound to the protein; in both cases, it coordinates to the imidazole group of a solvent-exposed histidine, i.e., His 64 in the case of carbonic anhydrase (Figure 2), and His105 in the case of ferritin (a single Ru ion on each subunit, as shown in Figure 2).

Also, the NAMI-A type complex AziRu manifests a similar mode of interaction with model proteins [62]. These aspects are comparatively examined in a recent review paper by Montesarchio et al. [63].

Based on the results illustrated above, we can state that the mechanism of protein ruthenation induced by NAMI-A-type compounds has now been satisfactorily clarified at the molecular level in various model proteins, and seems to be largely conserved. Typically, degradation of the Ru(III) complex anion proceeds completely, with the concomitant release of all other original Ru ligands, so that a naked ruthenium ion is eventually bound to the protein at selected side chains—mainly the imidazole group of histidine or the carboxylate group of Asp or Glu.

##### 2.2.2.2. KP1019

**Figure 3:**
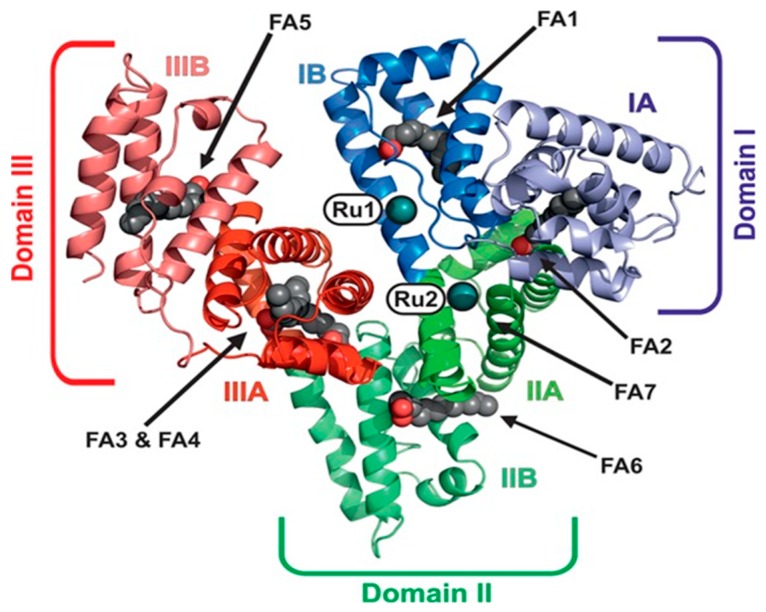
Overall structure of serum albumin (HSA)–Myr–KP1339 (Myr = myristate, domain I, blue; domain II, green; domain III, red). The two bound metal centers are labeled Ru1 and Ru2. The seven fatty acids (FA) bound to HSA are labeled as FA1 to 7 (aliphatic chain, gray spheres; carboxylate oxygens, red spheres). Reproduced from Ref. [65] (ACS AuthorChoice).

Unfortunately, work devoted to investigating the interactions of KP1019/KP1339 with model proteins usually failed to afford good quality crystals and the respective high-resolution crystal structures. For many years, the only available structural data was derived from a low-resolution crystal structure of the apo-lactoferrin adduct with KP1019 [64]. This study suggested that KP1019—at variance with NAMI-A—seems capable to retain one indazole ligand upon protein binding. The type of interaction consists in the coordination of ruthenium fragments to exposed histidine residues. However, a crystal structure of the HSA adducts with KP1019, although not at very high resolution, has now been reported by Keppler et al. [65], showing that—despite the greater inertness of KP1019 compared to NAMI-A—the dissociation of all ligands from the ruthenium center occurred also in this case, and two naked Ru ions were found coordinated to His146 and His242, which were both located within the hydrophobic binding pockets of albumin (Figure 3).

## 3. The Biological Profiles of NAMI-A and KP1019: Main Aspects

The main biological features of the two putative anticancer compounds have now been explored in depth through several studies carried out in vitro, at the cellular level, and in vivo, in a number of animal models. These studies are now integrated by the results of clinical investigations, some of which have appeared recently. In this section, we will survey the main results of these research activities to offer an overall and comparative biological description of both compounds.

### 3.1. In Vitro Cellular Studies

Since the very early studies, Sava et al. demonstrated that NAMI and NAMI-A-type complexes exhibit—in general—a negligible cytotoxicity that is unrelated to their antimetastatic activity [66]. The substantial lack of cytotoxicity for NAMI-A was later confirmed by other studies [67]. Notably, NAMI-A is, on average, more than 1000 times less cytotoxic than cisplatin against several tumor cell lines [68]; when tested in the 60-cell line panel of NCI for in vitro anticancer drug screening, it showed no activity [9]. In an indirect test performed on mice bearing Lewis lung carcinoma, NAMI-A was found to target primarily tumor cells endowed with metastatic ability within the primary tumor (see also below) [69]. However, it is worth stressing that a recent study reported a notable exception to the above-mentioned established concept, proving that NAMI-A is highly cytotoxic toward a panel of leukemia cell lines [70].

In contrast, KP1019 is moderately cytotoxic in vitro. For example, when tested against a panel of chemosensitive cell lines and their chemoresistant sublines, IC_50_ values in the range of 50 to 180 μM were measured [71]. When compared with its sodium salt, KP1339, in a few cancer cell lines, KP1019 tended to be moderately more cytotoxic (mean IC_50_ 93.1 μM for KP1019 versus 115.1 μM for KP1319) [72]. Nevertheless, the significant correlation between the cytotoxicity profiles suggests that the two complexes—as expected—share similar modes of action. Interestingly, for both compounds, no correlation between total Ru uptake and cytotoxicity was found [72]. Both KP1019 and KP1339 were found to be moderately cytotoxic (30–95 μM)—but more cytotoxic than cisplatin and etoposide—in colorectal carcinoma cells (SW480 and HT29) upon short-term exposure (24 h) and induced apoptosis predominantly by the intrinsic mitochondrial pathway. However, upon long-term exposure (72 h), cisplatin and etoposide became much more effective than the two Ru compounds [73].

More recently, KP1339 was investigated in more realistic three-dimensional cell culture systems (cancer cell spheroids), where it was less cytotoxic than in conventional two-dimensional cultures (e.g., for HCT116 cells, the IC_50_ was 136 ± 27 in the 2D model versus 244 ± 14 in the three-dimensional (3D) model) [74]. Similar IC_50_ values were obtained in hypoxic as well as non-hypoxic spheroids, in contrast with the often invoked “activation-by-reduction” hypothesis (see below), according to which Ru(III) compounds serve as prodrugs and are activated in the hypoxic environment of the solid tumors [3,10,23].

Studies concerning the antimetastatic ability of KP1019 in vitro gave controversial results. The complex revealed some anti-invasive activity in monolayer cultures of breast cancer cell lines, causing the significant reduction of cell migration and invasion [75]. However, KP1339 was later found to have no anti-invasive activity in the cell line HT1080, either in the spheroid model or in the transwell assay [74].

### 3.2. Animal Tests and Biodistribution

As mentioned above, NAMI-A was first tested in mice [29,30] and found to prevent impressively the development and growth of metastases generated by several solid tumor models, both in the lungs (Lewis lung carcinoma, MCa mammary carcinoma, TS/A mammary carcinoma, B16 melanoma and H460M2, a human NSCLC (non-small cell lung carcinoma) xenotransplanted into nude mice) and in the brain (P388 leukemia) [67,69,76,77,78,79]. The reduction of the number (from 40% to 100%) and weight (from 70% to 100%) of metastases led to a longer survival of treated mice, and even to cures when combined with the surgical removal of the primary neoplasm [6,80]. The activity of NAMI-A seems selective toward metastasis, as no significant inhibition of primary tumor growth is observed. This difference cannot be ascribed to the pharmacokinetics of the complex. In fact, when mice bearing the MCa mammary carcinoma were given NAMI-A through i.p. injection; ruthenium concentration in the lungs (comparable to that in the liver and kidneys) was ca. two to three times higher than in the solid tumor [35]. A similar ruthenium uptake by the primary tumor and host tissues was found in mice bearing Lewis lung carcinoma and treated i.p. with NAMI-A [81]. Even when NAMI-A was injected directly into the tumor mass, the reduction of the primary tumor growth was still modest compared to that of lung metastases, even though ruthenium concentration in the solid tumor was ca. one order of magnitude higher than in the lungs (where the concentration was similar to that obtained with i.p. treatment) [80,82]. It was also found that the decrease of ruthenium level from the lungs, liver, and kidneys is remarkably slower than from the primary tumor, suggesting a stronger binding and persistence in those tissues [82].

At variance, KP1019 was originally found to be highly active, with a tumor volume reduction up to 95%, in an autochthonous colorectal carcinoma of the rat that is platinum-resistant and resembles the colon cancer of humans (comparable histological appearance and behavior toward chemotherapeutics) [1,25,83]. In addition, KP1019 was tested in vitro against more than 50 primary tumors explanted from humans: in this highly predictive model, the complex afforded a positive response rate higher than 70% [84].

The time-dependent tissue distribution of KP1339 (given i.v.) in non-tumor-bearing BALB/c nude mice was recently determined [85]. The highest (and comparable) Ru concentrations were found in the liver, lungs, kidneys, and—surprisingly—in the thymus, followed by spleen and colon (ca. 50% less). Consistent with the trend of total Ru in blood plasma, the peak levels in the mentioned tissues were found one to six hours after administration and decreased slowly with time, with the exception of the spleen, where the highest amount was found 24 h post-injection.

### 3.3. Clinical Investigations

NAMI-A was introduced into a phase I study in 1999; it was the first ruthenium drug candidate to be tested in humans [86]. The study comprised 12 dose levels (2.4–500 mg/m^2^/day). Twenty-four adult patients, with different types of solid tumors, were infused with NAMI-A (3 h) for five consecutive days every three weeks. The toxicity profile of NAMI-A was quite different from that of the platinum anticancer drugs: mild renal toxicity, which was observed at the highest doses, was completely reversible, whereas hematological toxicity was negligible. Doses ≥400 mg/m^2^/day resulted in painful blisters on fingers and toes, which were dose-limiting. At the maximum tolerated dose (MTD) of 300 mg/m^2^/day, mild to moderate (and treatable) general malaise, nausea, vomiting, and diarrhea were observed. The total body retention of ruthenium was longer than expected from the preclinical studies [87], due to extensive binding to blood proteins.

No partial or complete responses were obtained, but disease stabilization (up to 21 weeks) was observed in heavily pretreated patients with advanced NSCLC. However, it should be remembered that efficacy is not the main objective of a phase-I trial, and that the majority of patients typically have a progressive, therapy-refractory disease, and have received multiple lines of treatment before recruitment.

By virtue of this result and of the activity shown by NAMI-A against lung metastases in mouse models (see above), and in analogy with the gemcitabine + cisplatin regimens widely used for first-line treatment of NSCLC [88], this cancer was selected as the target disease for a phase-I/II combination study of NAMI-A + gemcitabine. The study (2008–2011) was carried out on 32 patients with advanced NSCLC [89]. Gemcitabine was given at the typical dose of 1 g/m^2^ on, whereas dose escalation of NAMI-A—administered by i.v. infusion (3 h) on days 1 and 8 of a three-week cycle—was performed in phase I of the study. CTC (common terminology criteria) grade 2–4 neutropenia and anemia were observed at the highest doses, whereas the already mentioned blisters (dose-limiting toxicity) appeared at 600 mg/m^2^. The MTD was found to be 450 mg/m^2^, where the main non-hematological adverse events were elevated liver enzymes, transient creatinine elevation, renal toxicity, constipation, and fatigue. In phase II of the study, the antitumor activity according to RECIST (response evaluation criteria in solid tumors) criteria for solid tumors [90] was assessed on 15 NSCLC patients treated with the MTD of NAMI-A. A further expansion of the phase II cohort with additional patients was not pursued because the efficacy of the treatment turned out to be lower than expected for gemcitabine alone (one case of partial remission and 10 patients with stable disease for at least six to eight weeks). In addition, the patients found the combination treatment to be very exhausting, mainly because of the quite severe nausea, vomiting, and diarrhea. In conclusion, the treatment was declared to be “insufficiently effective for further use” [89].

In 2006, a preliminary phase I dose-escalation study was performed with KP1019 (total doses from 25 to 600 mg) on only eight patients with advanced solid tumors [2,91]. The complex, given i.v. twice a week over three weeks, was well tolerated in the investigated dose range, and only mild toxicity was observed [92]. Disease stabilization for eight to ten weeks, unrelated to the dose, was observed for five out of six evaluable patients.

In this study, the MTD of KP1019 could not be determined due to insufficient solubility (too large volume of infusion solution required for further dose escalation), and therefore, a full-scale phase I study was later performed on 34 patients with the more soluble sodium derivative Na[*trans*-RuCl_4_(Ind)_2_] (KP1339/IT-139). The investigation comprised nine dose levels (20–780 mg/m^2^/day), and the complex was given by i.v. infusion on days 1, 8, and 15 in a 28-day cycle [3]. Only minor adverse effects were observed. Grade 2–3 nausea together with increased creatinine levels were found to be DLT (dose limiting toxicity) at the highest dose. Stable disease (SD), up to 88 weeks, was found for seven patients with different types of tumors (including two cases of NSCLC), and one patient with a neuroendocrine tumor had a partial response (PR).

More recently, the phase I clinical investigation was repeated on 46 patients, with the same dose levels and treatment schedule, by Burris et al. in the United States (US) [93,94]. The MTD was established to be 625 mg/m^2^. Also, the tolerability and safety profile were similar to those prior established: no significant hematological toxicity or neurotoxicity were found, the main adverse events being clinically manageable grade ≤2 nausea, fatigue, and vomiting. Overall, the complex showed a moderate antitumor activity, with 26% disease control rate—that, interestingly, concerned three of the five patients with carcinoid neuroendocrine tumors—and one partial response in a patient with colon cancer.

## 4. Mechanism(s) of Action: Myths and Facts

Despite the numerous investigations carried out so far, we must admit that the mechanisms of action of NAMI-A and KP1019 are still largely unclear. This is not particularly surprising: even for the long established Pt anticancer drugs, there is still no general consensus on the actual molecular mechanisms. Indeed, the commonly accepted theory for the mode of action for cisplatin, which postulates its direct DNA binding and the consequent impairment of DNA functions (the so-called “DNA paradigm”), is now beginning to be questioned as too simplistic [95,96,97]. In addition, recent findings revealed profound mechanistic differences between two major platinum drugs, i.e., cisplatin and oxaliplatin [98].

In any case, some strong indications concerning the actual molecular mechanisms of NAMI-A and KP1019 may be certainly inferred from the large body of experimental results gathered on these compounds during the last two decades. For instance, Ru coordination compounds have a greater lability compared to Pt drugs, and this marks a relevant difference. As a matter of fact, the relatively fast aquation processes and ligand-exchange reactions of Ru compounds produce several species that are capable of reacting with a variety of biological components. This is particularly true for NAMI-A, which is far more labile than KP1019. This greater lability makes the associated metal activation and biomolecular metalation processes very complex upon considering the multitude of the resulting ruthenium-containing species that are formed in the biological media. It follows that a satisfactory description of the “true” mode of action of ruthenium compounds is extremely difficult to obtain, as the individual role of each ruthenium species should be identified and characterized as well as its specific contribution to the overall biological effect. This goal, at least in principle, might be partially achieved through a systematic implementation and integration of new “omics” techniques allowing a comprehensive interpretation of the obtained data in terms of the perturbation of the main signaling and metabolic pathways of the cells. Some preliminary results in this direction are just starting to be obtained, as it will be described later in this paragraph.

On the other hand, we find it appropriate to remark that during the last two decades, a number of fascinating theories were formulated concerning the mechanism of action of ruthenium compounds.

The main ones are mentioned below:

- Ruthenium (III) compounds are activated by reduction (activation-by-reduction mechanism)
- Ruthenium mimics iron metabolism and, as a consequence….
- …Transferrin is a selective carrier for ruthenium-based drugs, and….
- …Ruthenium compounds are not toxic
- DNA is the main target for ruthenium drugs.

However, even though these concepts may be charming and attractive, and reasonably grounded on known aspects of ruthenium chemistry, they could not be validated in the investigated biological systems; instead, they were often shown to be, at least partially, false and misleading, in particular when propagated and amplified by review articles, even very recently [43,99,100].

For example, recent XANES studies performed on KP1339 in several tissues (tumor included) of a SW480-bearing mouse suggest that the complex remains in its +III oxidation state after 24 h [101], which is in clear contradiction with the so-called “activation by reduction” mechanism.

Also, the idea that the iron transport protein transferrin might act as a selective carrier of ruthenium drugs to cancer tissues, although long cultivated and still cited in the current literature as a valid assumption, was not proven. Indeed, consistent with the relative concentration of transferrin in the blood, experimental evidence suggests that the transferrin adducts of these ruthenium compounds are not the major adducts formed in the blood after their injection. In the case of KP1019, it was found that the percentage of complex associated to transferrin is—at best—only a very small fraction of the total administered [102,103,104]. Similarly, in the case of NAMI-A, a recent study by Spiewak and Brindell demonstrated that transferrin is not the major binding partner for the complex [105]. In addition, no conclusive evidence has ever been reported on the circumstance that the transferrin adducts of these drugs may bring a significant pharmacological advantage over the free drug; more often, the association of ruthenium drugs to transferrin seems to result in a strong attenuation of their pharmacological action.

Thus, at present, the above theories and concepts must be considered mostly as myths: accordingly, they must be handled with extreme caution and possibly discarded and/or overcome to avoid their negative influence in the “correct” interpretation of new experimental results. In fact, it is evident that these concepts largely dominated the scenario of mechanistic research on ruthenium-based drugs during the last two decades and somehow hampered its evolution. An extensive critical discussion of these issues may be found in ref. [10].

Conversely, after 30 years of studies, we can enumerate a number of facts, well-grounded on experimental evidence, that now seem well established:

- It is true that NAMI-A and KP1019/1339 are formally very similar. However, there are some apparently small but important chemical differences that heavily affect their respective reactivities and thus their biological profiles.
- Both Ru compounds are not very stable in physiological media, with NAMI-A being significantly less stable than KP1019. Upon considering their moderate stability under physiological conditions, both Ru drugs may be straightforwardly classified as prodrugs.
- Both compounds are sufficiently water-soluble and may be used as such.
- Both compounds enter the bloodstream and associate strongly to plasma proteins, in particular to serum albumin. In spite of that, substantial amounts of ruthenium distribute to the whole body and reach several body compartments in an effective way.
- Both compounds manifest a relatively low degree of systemic toxicity and may be used quite safely up to relevant concentrations.
- The pharmacological profile of the two is highly distinct: KP1019 still produces an appreciable cytotoxic effect, while NAMI-A mainly causes antimetastatic effects.
- The different behavior is probably mediated by their different interactions with cells. Indeed, KP1019 is able to enter cells in appreciable amounts, whereas NAMI-A mostly localizes extracellularly or on the cell membrane, this being in our opinion a very crucial mechanistic distinction.

On the basis of the above facts and of the considerable number of cellular studies now available, we may also try to draft some realistic hypotheses concerning their respective mechanisms of action and underscore the main differences, as detailed below.

### 4.1. NAMI-A

At present, the pharmacological action of NAMI-A seems to be the result of a variety of concurrent mechanisms that apparently do not involve nuclear DNA [9,14]. Pioneering studies by Sava et al. demonstrated years ago that NAMI-A selectively affects tumor cells with metastatic ability within the primary tumor (Lewis lung carcinoma) [77], but scarcely affects the primary tumor itself. Several in vitro investigations on different cancer cell lines by the groups of Sava [77,78,106,107,108] and Lay [56] confirmed the capability of NAMI-A to affect significantly tumor cells with metastatic ability by interfering—at sub-cytotoxic and physiologically relevant Ru concentrations—with important steps of the tumor metastatic progression. Some more recent observations are in line with this hypothesis. Notably, by virtue of its relatively fast ligand-exchange kinetics, NAMI-A is not significantly internalized by cells. This seems to be a major mechanistic difference compared to KP1019, which is internalized by cells in far greater amounts. The difference in ruthenium uptake was further and robustly supported in a comparative study performed by Harris et al. based on the X-ray fluorescence imaging of single cells [109].

Thus, the biological effects of NAMI-A seemingly derive from ruthenium binding to collagens of the extracellular matrix and cell surface integrins, leading to increased adhesion and the reduced invasiveness of cancer cells. In turn, the activity of NAMI-A against already grown metastases is perhaps more reasonably attributable to its anti-angiogenic properties, which were confirmed in the chick chorioallantoic membrane and in the rabbit eye cornea model [110,111]. Conversely, the modest cellular uptake of ruthenium is in nice agreement with its scarce effects at the cytosolic level and its low cytotoxicity.

### 4.2. KP1019

There is now a considerable body of evidence pointing out for KP1019 a mode of action that is very distinct from that of NAMI-A [21]. These differences probably arise from the observed kinetic differences in the aquation processes and ruthenium activation. A key point in our opinion is again represented by the large difference in ruthenium uptake in the two cases leading to significantly higher ruthenium concentrations in the cytosol for KP1019. Accordingly, the in vivo activity of KP1019 on primary tumor growth is believed to be predominantly due to cytotoxic effects on tumor cells arising from a direct interference with cell signaling and metabolic pathways; in other words, KP1019 behaves as a classical cytotoxic drug. More precisely, one of the most recent and credited interpretations of the molecular mechanism of KP1339 tends to rule out a direct DNA damage as the main determinant of its cytotoxic action. In contrast, the postulated mode of action involves strong interactions with cytosol proteins leading to reactive oxygen species (ROS) overproduction, oxidative stress, and ER (endoplasmic reticulum) stress through targeting the chaperone protein GRP78. Eventually, this cellular damage triggers apoptosis through a mitochondrial pathway [112]. However, such mechanisms may be only a part of a more complex scenario.

**Figure 4:**
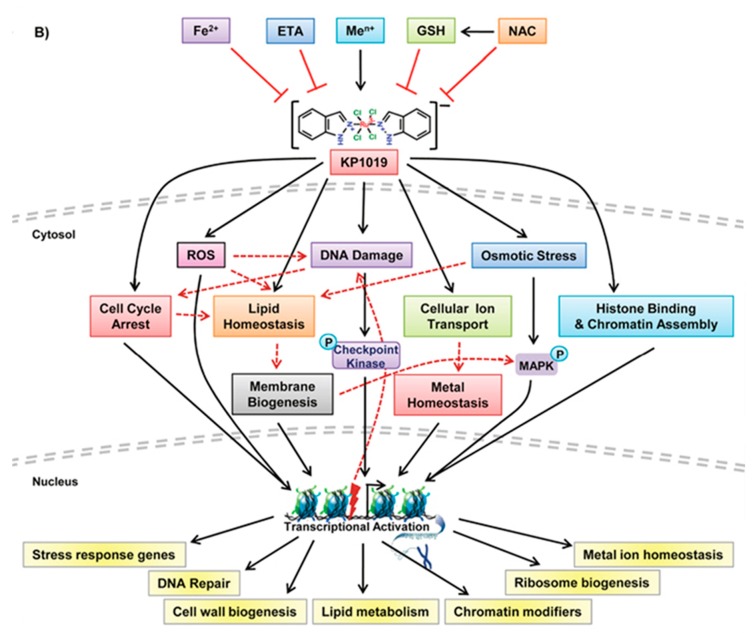
A proposed model depicting the mode of action of KP1019. Reproduced from Ref. [113].

In this respect, it is worth mentioning a very recent study by Tomar et al. [113]. Based on a quite complex investigative strategy relying on transcriptomics and a genetic screening approach in a budding yeast model, these authors identified various genetic targets and a plethora of cellular pathways targeted by KP1019. Then, the actions produced by KP1019 in yeast were compared with those produced by the same Ru drug in Hela cells, and reasonable extrapolations were made. On the ground of the obtained results, a comprehensive model depicting the mode of action of KP1019 and the targeted cellular pathways was proposed. According to this model, KP1019 induces ROS generation, causes DNA damage and thus cell cycle arrest, activates MAP (mitogen-activated protein) kinase signaling, alters intracellular metal ion and lipid homeostasis, and also affects the chromatin assembly. Cells activate transcriptional responses to alleviate cellular damage (see Figure 4).

Moreover, it was found that the toxicity potential of KP1019 is enhanced in the presence of various metal ions but suppressed by the supplementation of Fe^2+^ ions, and reduced glutathione (GSH), *N*-acetylcysteine (NAC), and ethanolamine (ETA). Thus, this model postulates for KP1019 a realistic multifactorial mechanism mediated by a variety of yet unknown molecular targets.

## 5. Conclusions and Perspectives

The development of NAMI-A and KP1019/KP1339 is an instructive case story in the field of medicinal inorganic chemistry. Thirty years of intense investigations have produced a lot of information on these two compounds, and also contributed to their introduction into clinical studies. Independently of the eventual clinical success of these two compounds, this story teaches us quite a few things.

NAMI-A and KP1019/KP1339 are suited for pharmacological investigations and pharmacological testing, although their stability is not high, and they undergo facile chemical transformations. Their behavior is typical of classical prodrugs, and similar to that of cisplatin and related platinum drugs. In addition, these compounds manifest an acceptable solubility in biological fluids, and their toxicity is limited and tolerable.

The fact that both compounds have been investigated in clinical trials producing scarce evidence of systemic toxicity increases their chances of clinical use. Even though their anticancer effects seem to be rather limited when they are used as standalone agents, there is still the chance to explore a larger number of cancer models and also use these compounds in combination therapies.

The interest in these ruthenium drugs has generated a lot of scientific publications. However, all this material must be critically evaluated: particular attention must be paid to separate ideas, concepts, and theories that have not been validated (myths) from those that have found a solid experimental support (facts).

The mechanistic aspects have not been fully understood yet; however, it seems very likely that these ruthenium compounds possess a multifactorial and multitarget mode of action. In any case, the different cellular uptake of the two Ru compounds has now been highlighted, and seems to play a crucial and distinctive role. There is still a lot of space for new studies and for a complete understanding, in particular taking advantage of new omics technologies. In this frame, the mechanistic differences between NAMI-A and KP1019 as well as their respective targets should be better identified and elucidated.

Finally, this story may turn out to be very useful for the design of better ruthenium anticancer agents and for the preparation of chimeric molecules containing ruthenium fragments.
